# In a squeeze: Epibiosis may affect the distribution of kelp forests

**DOI:** 10.1002/ece3.4967

**Published:** 2019-02-19

**Authors:** Guri Sogn Andersen, Frithjof E. Moy, Hartvig Christie

**Affiliations:** ^1^ Department of Biosciences University of Oslo Oslo Norway; ^2^ Norwegian Institute for Water Research Oslo Norway; ^3^ Institute of Marine Research His Norway

**Keywords:** aquatic plant ecology, epibiont, epibiosis, growth, habitat loss, kelp forest, light, Norway, recovery, *Saccharina latissima*, Skagerrak, survival, temperature

## Abstract

The processes limiting the population recovery of the kelp *Saccharina latissima* after recent large‐scale loss from the south coast of Norway are poorly understood. Previous investigations do, however, suggest that the impacts of biotic interactions (epibiosis and competition) and increased water turbidity are important. We investigated the depth‐related patterns of growth, epibiosis, and mortality in two sample populations of kelp, from the south and the southwest coast of Norway. The investigations were performed over a period of seven months, in a crossed translocational study, where kelps were mounted on rigs at six depths (1, 3, 6, 9, 15, and 24 m). In a second experiment, the amounts of light blocked by different epibiont layers growing on the kelp frond were investigated. While growth decreased with depth in spring and summer, the kelp grew faster at 15 m than at shallower depths in fall. Survival was low both in shallow water and below 15 m depth. Epibionts covered the kelp growing at depths from 1 to 9 m, and the laboratory study showed that the coverage may have deprived the individuals of as much as 90% of the available light. Although the depth‐related results we present apply—in the strictest sense—only to kelp translocated on rigs, we argue that the relative patterns are relevant for natural populations. Growth and survival of *S. latissima* is likely to be reduced by heavy loads of epibionts, while depths where epibionts are sparse may be close to the lower limit of the kelps depth distribution along the south coast of Norway. This suggests that a vertical squeeze, or narrowing of the distribution range of kelp forests may be occurring in Norway.

## 1 INTRODUCTION

Sea grasses and seaweeds, including kelp species, provide important ecosystem services in coastal areas, and large‐scale losses of these macrophytes are a global concern (Araújo et al., 2016; Filbee‐Dexter & Wernberg, 2018; Harley et al., 2012; Krumhansl et al., 2016; Merzouk & Johnson, 2011; Müller, Laepple, Bartsch, & Wiencke, 2009; Nyström et al., 2012; Waycott et al., 2009; Wernberg, Russell, & Moore, 2011). Increased eutrophication in coastal areas (Eriksson, Johansson, & Snoeijs, 2002; Gorman & Connell, 2009; Waycott et al., 2009), changes in key species, and interactions across trophic levels (Baden, Emanuelsson, Pihl, Svensson, & Åberg, 2012; Fagerli, Norderhaug, Christie, Pedersen, & Fredriksen, 2014; Moksnes, Gullström, Tryman, & Baden, 2008; Pinnegar et al., 2000; Rinde et al., 2014; Sivertsen, 2006), climatic changes (Connell & Russell, 2010; Hiscock, Southward, Tittley, & Hawkins, 2004; Wernberg et al., 2016, 2012), or additive and synergistic combinations of several of these factors (Baden et al., 2012; Burkepile & Hay, 2006; Filbee‐Dexter, Feehan, & Scheibling, 2016; Harley et al., 2012; Jackson, 2008; Ling, Johnson, Frusher, & Ridgway, 2009; Provost et al., 2017; Strain, Thomson, Micheli, Mancuso, & Airoldi, 2014; Wernberg et al., 2011) have all been considered important drivers of recent losses.

*Saccharina latissima* (Linnaeus) C.E. Lane, C. Mayes, Druehl, and G.W. Saunders is a kelp species. Its populations are often dense and form underwater forest landscapes that provide habitats for myriads of species. *S. latissima* used to dominate in subtidal and sheltered areas along rocky parts of the Norwegian south coast, but disappeared sometime in the late 1990s (Moy & Christie, 2012). Because this kelp is a cold‐temperate water species (Müller et al., 2009), and unusually high sea water temperatures were recorded several summers during the late 1990s and early 2000s, heat stress may have been the cause of the *S. latissima* forest demise. After the heat waves, the temperatures were normal for several years. In this period, regrowth was probably not hindered by high sea water temperature (see, e.g. Sogn Andersen, Foldager Pedersen, & Nielsen, 2013).

Recent surveys have shown that a benthic community shift occurred when the kelp disappeared, resulting in complete dominance of filamentous red and brown algae (turf algae; Moy & Christie, 2012). Moy and Christie (2012) reported that healthy *S. latissima* populations still remained in wave‐exposed areas, and these should have been able to disperse and recolonize adjacent, deforested areas. Rapid forest recovery has occurred after large‐scale disturbances in the past, indicating that recolonization by this species used to be effective (Moy & Christie, 2012). At present however, the turf algae communities seem persistent along most of the south coast, and there are currently no signs of kelp forest recovery in the majority of the deforested areas (Moy & Christie, 2012). The importance of competitive interactions has been documented in kelp forests (Falkenberg, Russell, & Connell, 2012; Filbee‐Dexter & Wernberg, 2018; Gorman & Connell, 2009), but in the case of *S. latissima* forests in Norway, such interactions are poorly studied.

The strait running between the south east coast of Norway, the south west coast of Sweden, and the Jutland peninsula of Denmark is called Skagerrak. As in many coastal areas around the world, the water in Skagerrak has become increasingly turbid during the past decades (i.e., darkening of the water; Cossellu & Nordberg, 2010; Frigstad, Andersen, Hessen, Jeansson, & Skogen, 2013), and the depth to which sunlight can penetrate has therefore been reduced. A substantial change in the vertical distribution of photosynthesizing species (including *S. latissima*) has also occurred in Skagerrak over time (Eriksson et al., 2002; Moy & Christie, 2012; Pedersén & Snoeijs, 2001; Rueness & Fredriksen, 1991), and these changes have been coupled to the reductions in light availability.

In addition to the increased water turbidity, extensive epibiosis seems to be an increasing problem, and epibionts may deprive their host algae of much light. The effect of epibiosis on kelp is relatively poorly known (see however Lee & Brinkhuis, 1988, Levin, Coyer, Petrik, & Good, 2002, Hepburn & Hurd, 2005, Hepburn, Hurd, & Frew, 2006, Saunders & Metaxas, 2007, Scheibling & Gagnon, 2009), but Sogn Andersen, Steen, Christie, Fredriksen, and Moy (2011) suggested that their impact may be important in understanding the kelp loss in Skagerrak.

*Saccharina latissima* forests also deteriorated along the west coast of Norway in the late 1990s (Moy & Christie, 2012). However, the Norwegian monitoring programs have since documented a gradient of ecosystem recovery, from mainly disintegrated and lacking forests on the south east coast and in part on the south west coast to healthy forests in many areas on the mid‐west coast of Norway. This means that the west coast kelp have been able to disperse and recolonize while kelp in Skagerrak have not. The explanation for this may lie in environmental differences between the areas or in physiological differences between the local populations. Physiological traits in *S. latissima* populations may vary geographically (e.g., different environments may lead to adaptations resulting in ecotypic differentiation; Gerard, 1988, 1997; Gerard & Du Bois, 1988), and kelp individuals from different parts of Norway may therefore respond to stressors like low light and extreme temperatures in different ways. Temperature responses were tested in a recent study, and individuals from the intermediate and both extremities of the Norwegian gradient (south east to west) showed very similar photophysiological responses to temperature stresses (Sogn Andersen et al., 2013). The question of whether the tolerances differ when the kelp plants are subjected to the whole range of stressors encountered in situ is, however, not yet answered.

The present study investigated the relationship between depth and patterns of growth and survival of kelp from the south and west coast of Norway. These relationships were investigated in two areas: one site on the west coast representing an area in which kelp forests have been able to recover, and another site in the southeastern (Skagerrak) part of Norway, where recovery has been poor and kelp forests are mostly scarce. In a crossed experiment, kelp plants from both areas were translocated on rigs and monitored at six depths for seven months. The main objective was to establish whether kelp from the two populations responded in different ways.

Secondly, we investigated the extent of shading caused by different forms of epibionts that are commonly found on *S. latissima* in Skagerrak. The amount of shading was compared to the light reductions measured with increasing water depth on the Skagerrak site. This comparison was used in combination with the investigations of growth and survival, to indicate the impact epibiont shading may have had on the kelp. Particular attention was therefore given to the lower depth limit of *S. latissima*. We hypothesize that epibionts deprive their host of light and that the deprivation may become lethal.

## 2 MATERIALS AND METHODS

### 2.1 Growth and survival

**Figure 1:**
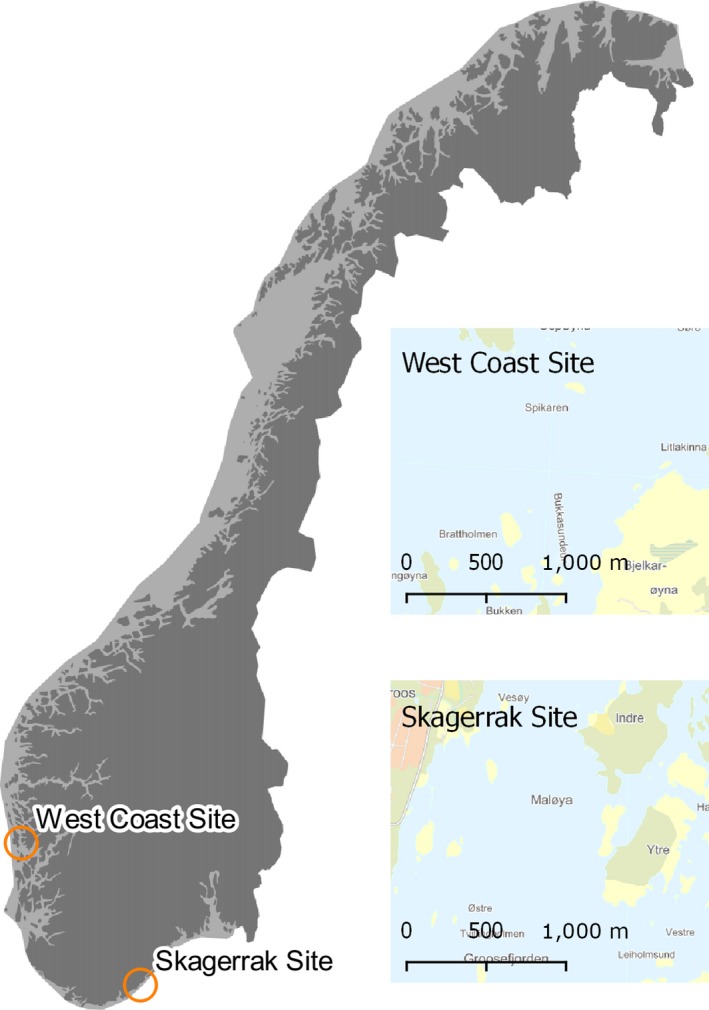
Experimental sites. Map showing Norway and the experimental sites

Two areas (hereafter called experimental sites) were chosen to represent the Skagerrak (south eastern) and the west coast (western) parts of what might be a *S. latissima* recovery gradient in Norway. The experimental site in Skagerrak was located at 58°19’N, 8°35’E (WGS84 datum) nearby Grimstad, while the experimental site on the west coast was located at 60°15’N, 5°12’E (WGS84 datum) nearby Bergen (Figure 1). The two areas had water depths of approximately 30 m and were classified as sheltered according to the wave exposure model developed by Isæus (2004).

At both experimental sites, four stations were picked for the deployment of the experimental rigs (eight locations in total). Each station was separated from other stations by more than 500 m.

Adult *S. latissima* sporophytes were collected at 6 m depth within a radius of 2 km from each of the experimental sites in February 2009. Sheltered sites within Skagerrak (i.e., exposed to the same level of wave action as the experimental sites) are still largely devoid of kelp, and kelp plants had to be sampled in more exposed areas. On the west coast, the sampling site was sheltered (same level of exposure as the experimental site). For simplicity, kelp sampled on the Skagerrak coast will hereafter be referred to as SCK, while kelp sampled on the west coast will be referred to as WCK.

Half of the kelp plants sampled were transported to the opposite coast so that each experimental site had both native and transported samples. The samples were transported in sea water and contained in dark transport coolers that kept the temperature low (5°C).

**Figure 2:**
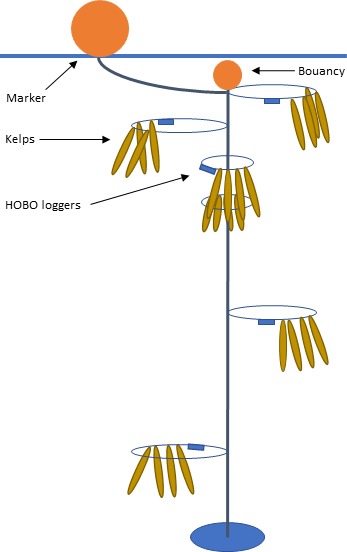
Rig. Schematic presentation of the rig setup

Kelp from different sampling sites (WCK or SCK) were mounted on separate rigs. On each rig, four mature kelp individuals (lengths of approximately 1 m) were attached at each of the six depths: 1, 3, 6, 9, 15, and 24 m (see Figure 2). At each station two rigs, one with WCK and one SCK were deployed, 20–30 m apart from each other. Thus, at each experimental site, eight rigs were monitored (384 kelp individuals in total).

In experiments where organisms are handled, method control should be applied in order to separate the effect of the handling from the effect of the surrounding environment. For proper control of the rig treatment, it would have been necessary to measure responses in individuals in natural populations at both sites and compare these results to the responses measured in individuals mounted on rigs within each site and at the same depths. Since the sites were largely void of natural populations at the time, this was impossible. As a suboptimal approach, treatment control could have been conducted within the more exposed sampling sites. This would have expanded the amount of work done by SCUBA diving in wave‐exposed areas, and the logistics required to execute this safely was not feasible within our project. However, our main objective was to test the relative effect of depth on growth and survival. With this in mind, the use of rigs provides great benefits because it ensures as similar conditions between the stations as possible. The monitoring was continued for seven months, and the effects observed late in the study were less likely to be caused by the translocation per se. Our results apply thus, in the very strictest sense, only to translocated kelp on rigs. However, we argue that the relative differences among kelp plants mounted at the different depths still provide insight to the effects that can be expected in natural populations.

The frond length and frond width of each kelp plant were measured at the initiation of the experiment. A small hole was punched 10 cm above the transition zone, between stipe and frond, to be able to measure elongation according to the method described in Fortes and Lüning (1980). Growth (*G*_rate_) as rate of daily areal increase was calculated according to formula 1:

(1) $$G_{\text{rate}}∼\frac{2EW_{1}}{\left(L\left(t\right)W_{2}\left(t\right)+\left(L\left(t-1\right)+E\right)W_{2}\left(t-1\right)d\right)},$$

where *L* and *E* are the total length and elongation of the lamina, respectively, *W*_1_ and *W*_2_ are the lamina widths 10 and 50 cm above the basis respectively, *t* is the point of measuring, and *d* is the number of days since the last measuring event.

The extent of epibiont coverage on the kelp frond (in percentages) was noted throughout the experiment. Survival was recorded as a binary response, and kelp individuals that had been torn off and individuals with severely perforated and bleached meristems were recorded as dead.

The rigs were monitored for seven months (February 2009 to September 2009), and measurements were executed in spring, summer, and fall according to Table 1.

**Table 1:** Dates (2009) in which measurements were executed. The rigs on the west coast were lost in fall, probably due to a combination of boat traffic and bad weather

Location	Initiation	Spring	Summer	Fall
Skagerrak	February 22nd	March 26th	May 19th	September 25th
West coast	February 20th	April 14th	June 10th	Rigs lost

The relationships between growth in the sample populations (WCK and SCK), depth, and season at both experimental sites were investigated. Because growth was measured as percentage change in size, the response was log‐transformed and analyzed assuming normal errors (see chapter 28 in Crawley, 2002, page 514). The statistical analyses were performed using the protocol of Zuur et al. (see chapter 5 in Zuur, Ieno, Walker, Saveliev, & Smith, 2009, page 127) for mixed linear modeling, first including all explanatory variables (Site, Population, Depth, and Season) and their interactions. Because spatial autocorrelation was likely to occur, we could not assume that the response (growth) was independent within a station. Station was therefore included as a random factor in the model selection process. The Bayesian information criterion (BIC) was used in order to select between model alternatives (model selection step 1), and backwards model selection by successive likelihood ratio tests was used in excluding parameters (selection step 2; Zuur et al., 2009). Finally, the variance structures (i.e., normality and homogeneity of errors) were evaluated by visual inspection of residual plots (Zuur et al., 2009).

Upon inspecting the model residuals, both depth‐ and season‐dependent error structures were revealed, and these dependencies violated the underlying assumption of linear models meaning that we could not trust the *p*‐values. A second‐degree polynomial term was therefore added to improve model fit with depth. An additional weights term (varIdent in the *nlme* package of R) was also added, to allow for seasonal differences in variance (see chapter 4 in Zuur et al., 2009). These measures dealt with the issues of heteroscedasticity (selection step 3).

We also investigated the patterns of epibiont coverage and survival by the end of the experiment. The relationships between the percent cover of epibionts, survival (success or not), and depth in both WCK and SCK were tested by the use of generalized linear mixed modeling with binomial distributions. Station was included as a random factor in both models for the same reason as mentioned above.

We used the R computer software (R Core Team, 2013) for all computations. In the statistical analyses, we used the *nlme* (Pinheiro, Bates, DebRoy, Sarkar, & R Core Team, 2013) and *lme4* (Bates, Maechler, & Bolker, 2013) R packages.

### 2.2 Ambient temperature and light

Temperature data were retrieved from the operational ocean forecast database at the Norwegian Meteorological Institute.

The ambient light condition in the water column was recorded throughout the experiment. Small, light logging HOBO pendants (UA‐002‐08, Onset Computer Corporation, USA) were mounted at five depths (3, 6, 9, 15, and 24 m) directly above the kelp holdfasts on two rigs within both experimental sites (20 loggers in total). HOBO pendants measure light at wavelengths from approximately 150 to 1,200 nm. Within this range, the sensitivity of the sensor differs, and in the range that covers photosynthetically active radiation (PAR, 400–700 nm), it is below 80%, which contributes to an underestimation of total light hitting the sensor. The sensitivity is much lower in the ultraviolet (UV) parts of the spectrum (<40%) but reaches an optimum of close to 100% within the infrared (IR) parts of the spectrum. In seawater however, almost all light in the IR zone is absorbed within a depth of 1 m. And although UV radiation penetrates deeper, merely 8% of the sunlight that hits the ocean surface is UV radiation. The contribution of IR and UV radiation compared to PAR in our measurements is therefore very small. For the purpose of the here presented study, we consider the HOBO measurements a meaningful (although underestimated) approximation of PAR (see Long, Rheuban, Berg, & Zieman, 2012), in concurrence with other studies within the field of kelp forest ecology (Bennett et al., 2015).

During the measuring event, the pendants were wiped clean of fouling organisms that could affect the sensors. At the termination of the experiment, the pendants were brought back to the laboratory, and data from seven days following each cleaning were extracted and pooled.

The rigs with light loggers were unfortunately lost from the west coast site.

### 2.3 Shading by epibionts

Fifty kelp plants were harvested (at approx. 6 m depth) from one of the few remaining forest patches in Skagerrak in October 2009. The harvest site was located close to the sample site used in the rig experiment.

Dominating epibionts found in Skagerrak are vase tunicates (*Ciona intestinalis*), encrusting bryozoans and filamentous algae (mostly red algae; Moy & Christie, 2012; Sogn Andersen et al., 2011). All of these were present on the individuals we harvested. Vase tunicates and bryozoans form colonies that are almost uniform in density, while the densities of the epiphytic algal layers vary considerably. Vase tunicates and bryozoans were therefore regarded as either present or absent, while the densities of the algal epiphytes were measured in dry weight per substrate (kelp sample) area (DW/cm^2^). Forty‐four samples were taken from the fifty kelp individuals in order to estimate algal densities and perform the light measurements.

To estimate the amount of shading caused by epibionts, a series of light measurements were performed. We used a TriOS RAMSES (TriOS Optical Sensors, Germany) with 198 channels to measure light in the range of wavelengths from 310 to 950 nm. The sensor was connected to a field‐PC with MSDA software to record the readings. A cylinder of plexi‐glas served as a measuring chamber, and a lamp (FieldCal) that was fitted on top of the measuring chamber served as the light source. Each measurement was repeated three times, and the values were averaged. This was done in order to reduce the influence of noise that could occur in the readings (judged by previous experience with the equipment).

The measuring chamber was filled with sea water (~10°C). A kelp sample was then cut from a harvested individual with a cork borer and fitted into the measuring chamber. The extent of shading caused by each type of epibiont (either vase tunicate, bryozoan, or algal coverage) was estimated from nine replicate samples. In addition, we performed a series of measurements in which varying amounts of epiphytic algae were transplanted on top of a kelp sample. In this case, epibionts from the harvest was used to ensure a realistic composition of algal species. This additional step was done in order to further investigate the relationship between the density of algal epiphytes and shading.

To estimate the light deprivation caused by epibionts, we had to compare light measurements from samples *with* epibiont coverage to measurements *without* them. The latter will hereafter be called the reference readings. In the measuring process, the reference readings had to be obtained in different ways. In the case of vase tunicates, the animals were gently removed from each sample before the reference readings were performed. Bryozoan crusts and filamentous algae on the other hand were impossible to remove without scarring the kelp lamina, and scars would have affected the light measurements. In these cases, clean tissue samples (from each kelp) were therefore taken to serve as references.

The amount of light that penetrated each sample was estimated by calculating the integral of the light intensities measured in all wavelengths. The integrals were calculated by the trapezoidal rule approximation method and the amount of shading was estimated according to formula 2.

(2) $$\int_{a}^{b}f\left(x\right)dx-\int_{a}^{b}g\left(x\right)dx,$$

in which *x* is wavelength, *a* = 310 nm, *b* = 950 nm, and *f* and *g* are the curves describing light penetrating profile in the reference and samples covered by epibionts, respectively.

The effect of either vase tunicate or bryozoan presence (i.e., presence or absence) on shading was analyzed using a generalized linear model with a binomial distribution. In a separate analysis, the effect of different algal densities on shading was described by an asymptotic function fitted by nonlinear least‐square regression. For both analyses, we used the *stats* (R Core Team, 2013) R package.

## 3 RESULTS

### 3.1 Growth

The rigs on the west coast were unfortunately lost before fall. We do not know the cause, but there was some bad weather with lots of wind in late summer/early fall which may have moved the rigs and/or increased the chance of passing boats hitting them and severing the ropes. To compare growth between populations and between sites, we thus initially excluded data from fall. Once establishing that there were no site‐specific differences in growth rates for any of the sample populations, we continued the analyses using the full dataset. In analyzing the growth rates, several statistical models were built and compared (see Table 2), but in the end, a linear model, without random terms, was considered the best fit judging by successive likelihood ratio tests and because it obtained the lowest BIC value (Zuur et al., 2009; Table 3). The residuals did not show any trend with station (the random factor) which also indicated that mixed effect modeling was not required (see Zuur et al., 2009).

**Table 2:** Model selection in relation to growth. Both generalized least‐square models (*gls*) and linear mixed effect models (*lme*) were tested

Selection	Step 1			Step 2	Step 3		
Model	1a	1b	1c	2	3a	3b	Final
R function	*gls*	*lme*	*lme*	*gls*	*gls*	*gls*	*gls*
Site	x	x	x	x	x	x	
Pop	x	x	x	x	x	x	x
Depth	x	x	x	x	x	x	x
Depth^2^				x	x	x	x
Ssn (Season)	x	x	x	x	x	x	x
*Site* × *Pop*	x	x	x	x	x		
*Site* × *Depth*	x	x	x	x			
*Pop* × *Depth*	x	x	x	x			
*Pop* × *Ssn*	x	x	x	x			
*Ssn* × *Depth*	x	x	x	x	x	x	x
Random		Station	Station				
Random int		x					
Random slope		x	x				
Weights				Ssn	Ssn	Ssn	Ssn
Method		*REML*		*REML*		*ML*	
BIC	−4,852	−4,847	−4,841	−4,870	−5,115	−5,140	−5,144

Note

Parameters marked with an x were included in the models. Site and Pop annotate experimental site and sample population (WCK or SCK), respectively. In step 1, saturated models were fitted by maximizing the restricted log‐likelihood (REML). The model with the lowest BIC was chosen for further parameter selection. In step 2, heteroscedasticity was dealt with by including a second‐degree polynomial (Depth^2^) and an additional weights term to allow for different variances between seasons. Selection of parameters was performed in step 3, where models were fitted by maximizing log‐likelihood (ML) and terms were excluded in a stepwise manner using the likelihood ratio test. Only a subset of models are shown. The model with the lowest BIC was the best fit (Final). The final model was also refitted using REML

**Table 3:** Final growth model. Estimates from (a) the growth rate model with the best fit (see Table 2) and (b) a separate model excluding data from fall. The baselines (Intercept) in the Gaussian models were (a) growth rates in the west coast sample population (WCK) in Spring and (b) growth rates in WCK on the Skagerrak coast in Spring. Site was excluded in the best model, without influencing the parameter estimates much, in support of Site not influencing the growth rates

	Parameter	Estimate	*SE*	*t*	*p*
(a)	Intercept (WCK in Spring)	0.61501	0.013647	45.06471	<0.0001
	Population (SCK vs. WCK)	−0.01964	0.008718	−2.25289	0.0247
	Depth	−0.00007	0.002311	−0.02976	0.9763
	Depth^2^	−0.00091	0.000088	−10.34669	<0.0001
	Season (Summer vs. Spring)	−0.03097	0.014375	−2.15416	0.0317
	Season (Fall vs. Spring)	−0.60742	0.042574	−14.26738	<0.0001
	*Depth* × *Ssn* (*Sum vs. Spr*)	0.00615	0.001268	4.85206	<0.0001
	*Depth* × *Ssn* (*F vs. Spr*)	0.02923	0.003726	7.84604	<0.0001
Without data from fall					
(b)	Intercept (WCK in Skagerrak)	0.61508	0.013507	45.53817	<0.0001
	Population (SCK vs. WCK)	−0.01957	0.008977	−2.17963	0.0298
	Depth	−0.00010	0.002310	−0.04370	0.9652
	Depth^2^	−0.00091	0.000088	−10.28913	<0.0001
	Season (Summer vs. Spring)	−0.03098	0.014403	−2.15069	0.0320
	*Depth* × *Ssn (Sum vs. Spr)*	0.00615	0.001272	4.83782	<0.0001

**Figure 3:**
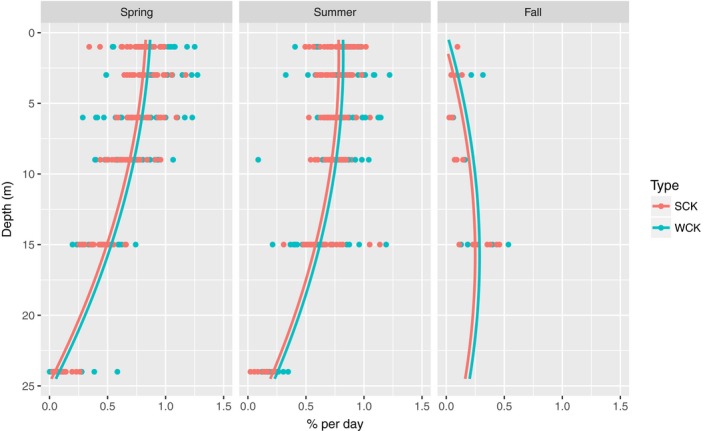
Growth. Seasonal growth rates in relation to depth. Site did not explain a significant part of the variation, and the results from the experimental sites were pooled in these presentations. Growth rates in SCK and WCK are represented by different colors. The lines (also different colors) are model predictions from the growth model (Table 3)

*Saccharina latissima* from the Skagerrak population (SCK) grew slightly slower than kelp from the west coast population (WCK) (Table 3 and Figure 3). The difference was consistent at all depths and throughout the experiment, which means that the response to depth treatment and season had been the same in both sample populations. The difference in growth between SCK and WCK was also consistent among the experimental sites, which indicated that transport and relocation had not affected these results.

The depth‐related pattern of growth changed from spring to fall (shown by the significant interaction between depth and season in Table 3). In spring and summer, growth generally decreased with depth (Figure 3 and Table 3). In fall however, the pattern was reversed in Skagerrak, and growth was faster at 15 m than at 3 m depth. All rigs in the west coast area were unfortunately lost by the end of the summer, and data from this area in fall could therefore not be retrieved.

### 3.2 Survival

**Figure 4:**
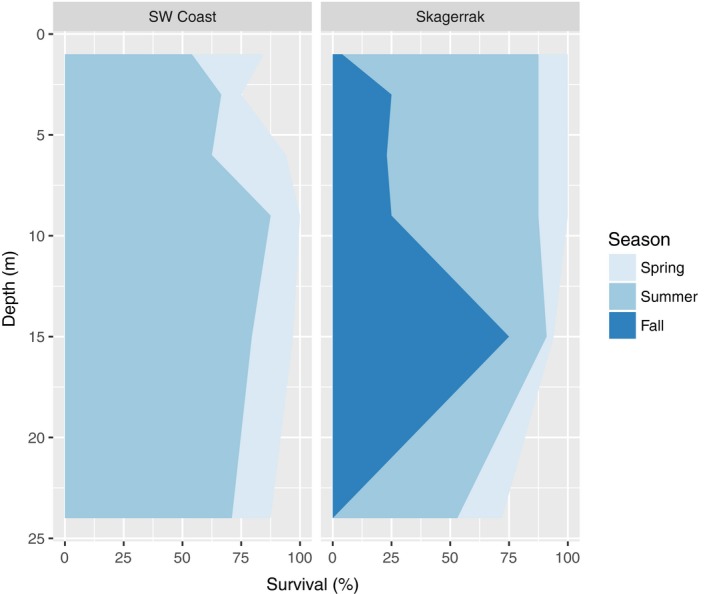
Survival. Kelp survival was recorded at the different sampling times in Skagerrak and on the west coast. The light blue area shows survival as recorded in spring, intermediate blue area shows survival as recorded until summer, and the dark blue area shows survival as recorded from the initiation of the experiment and until fall

Survival was analyzed in relation to depth. Since the west coast rigs were lost before fall, site‐specific differences were investigated using the summer data. Comparatively more kelp survived in shallow areas on Skagerrak site through spring and summer, while survival at 25 m depth was higher on the west coast site. When tested, these differences were, however, not statistically significant. By the end of the summer, most kelp plants had actually survived at all depths on both sites (Table 4 and Figure 4).

**Table 4:** Analysis of survival. Estimates from the analysis of survival on the west coast (in summer) and in Skagerrak (in fall). The baselines (Intercept) in the binomial models were in both cases survival in the west coast sample population. Station was included as a random factor

Parameter	Estimate	*SE*	*z*	*p*
Summer (on the west coast)				
Intercept (WCK)	15.86	93.84	0.169	0.866
Pop (SCK vs. WCK)	36.12	>100	0.000	1.000
Depth	~ 0	8.662	0.000	1.000
Depth^2^	~ 0	33.42	0.000	1.000
Fall (in Skagerrak)				
Intercept (WCK)	−3.722	0.811	−4.586	<0.001
Pop (SCK vs. WCK)	0.320	0.481	0.664	0.507
Depth	0.600	0.141	4.258	<0.001
Depth^2^	−0.024	0.006	−4.131	<0.001

**Figure 5:**
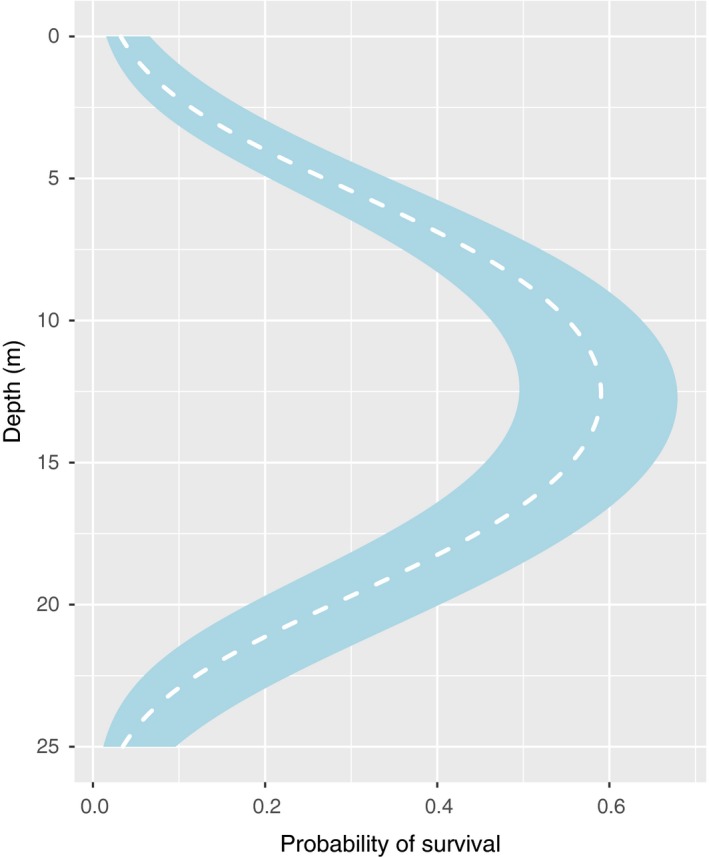
Probability of survival in fall. Predicted survival of *Saccharina latissima* on rigs in Skagerrak in fall (white, dashed line), according to the model presented in Table 4. The standard errors of the model predictions are represented by the ribbon (blue)

By fall, survival was very low both close to the surface and at 24 m depth in Skagerrak (<20%), but much higher at 15 m depth (70%–80%) (Figure 4). The model predicted an unimodal pattern of survival with the highest probabilities between 10 and 15 m depth in this area (Table 4 and Figure 5).

It was also important to find out whether the depth‐related pattern of survival differed between the two sample populations (WCK and SCK). The analyses showed no significant differences in survival between populations on either experimental site 4.

The kelp fronds were fouled by heavy loads of epibionts, and the general condition of the remaining kelp plants in fall was poor.

### 3.3 Temperature and light

**Figure 6:**
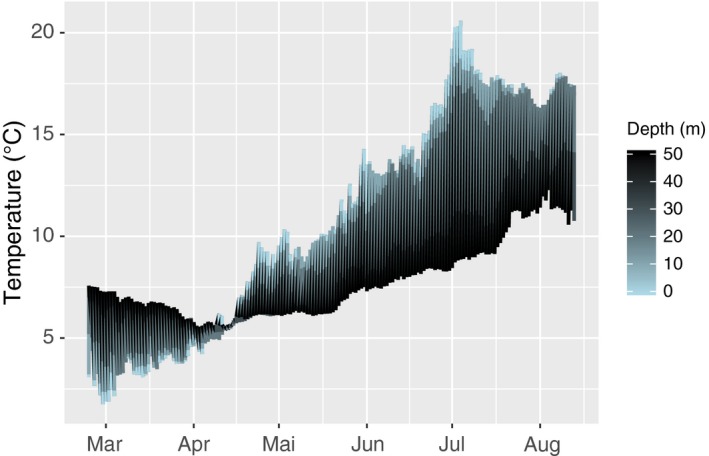
Temperatures. Temperatures from March to August 2009 at five depths as provided by the Norwegian Meteorological Institute

The daily means of ocean temperatures (at 0, 5, 10, 20, and 30 m depth) in Skagerrak from March to August 2009 are presented in Figure 6. The period with the highest sea water temperatures spanned from June (summer) to August (fall). The average sea water temperature at the surface was (17.6°C ± 0.2 *SE*) from July to August, and the difference down to 20 m depth was relatively small (−2.8°C ± 0.2 *SE*).

**Figure 7:**
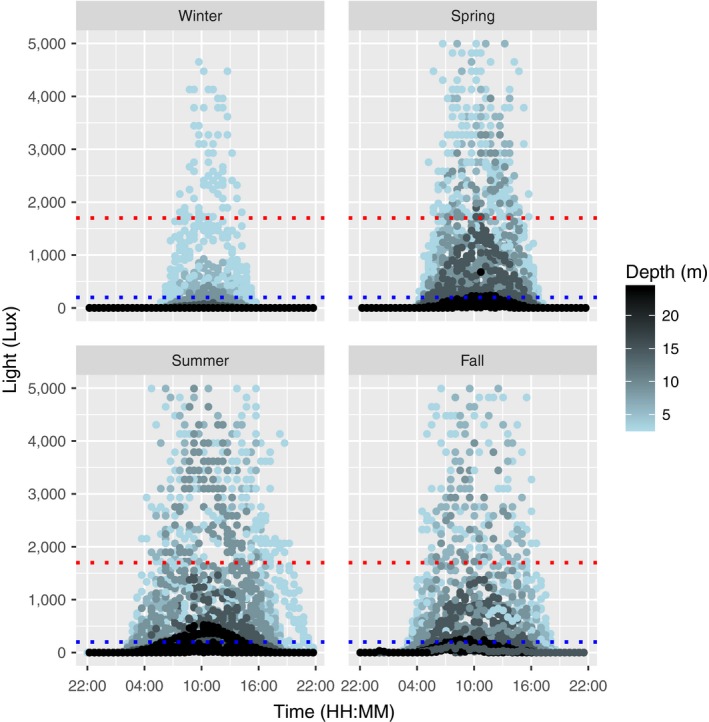
Light. Daily light dynamics in the water column (at 3, 6, 9, 15, and 24 m depth) in Skagerrak in through the seasons of 2009. Light intensities were measured by HOBO pendants. The dotted lines represent the compensation light intensities of *Saccharina latissima* grown at 10°C (lower) and 20°C (upper), respectively (according to Sogn Andersen et al., 2013)

**Figure 8:**
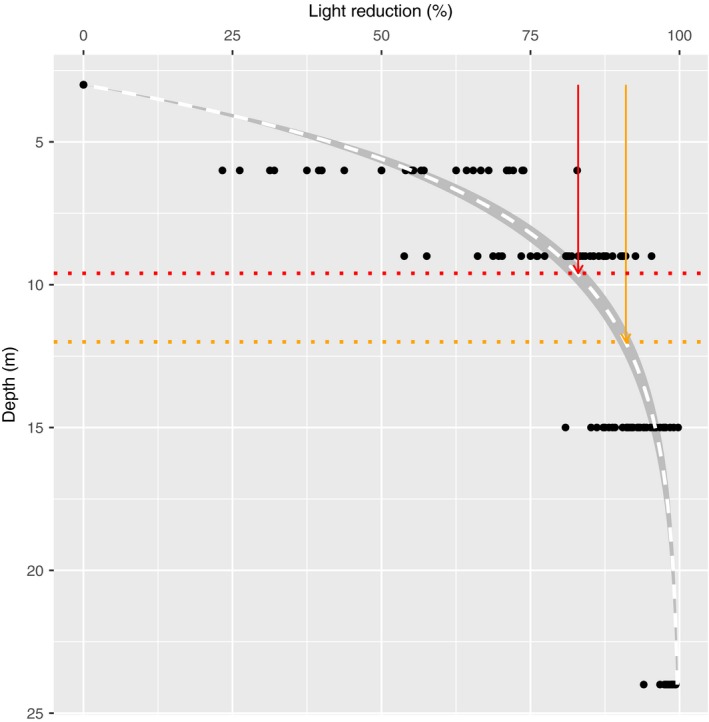
Light reduction with depth. Light reductions calculated from 3 m and down to 24 m depth in fall. The relationship between depth (*X*) and light reduction (*Y*) is described by the model, *Y* ~ 100(1 – *e* ^−0.26(^ *^X^* ^−3)^) (fitted by nonlinear least square regression), and represented by the dashed line (white). The shaded area depicts the 95% confidence interval of the model predictions. The vertical orange arrow indicates the light reduction experienced by a kelp at 3 m depth overgrown by a vase tunicate colony (*Ciona intestinalis*) (91%), while the red arrow indicates the light reduction experienced by a kelp at the same depth overgrown by the mean density of algal epiphytes found on naturally occurring kelp in Skagerrak in October (83%). The vertical dotted line indicates the depth “equivalents” with regard to light intensity (approximately 13 and 9 m depth, respectively)

The 24‐hr light dynamic schemes varied with depth and season (Figure 7). The shape of the curves was similar all seasons, while the intensity and the rate of reduction with depth varied due to seasonal changes in the irradiance and the solar elevation angle. In fall, light decreased rapidly with depth, and the intensity was reduced with between 80% and 100% at 15 m depth as compared to at 3 m depth (Figure 8).

### 3.4 Epibionts

In spring, kelp plants from both sample populations appeared clean and healthy in both areas. Scattered turfs of epiphytic algae and colonies of bryozoans were observed in both areas in summer, while vase tunicates were only observed in Skagerrak. The amounts were in every case too low to be estimated as coverage.

**Figure 9:**
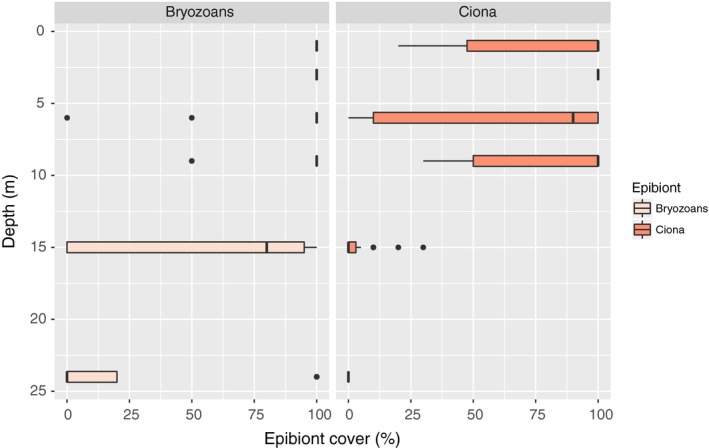
Epibiont cover. Percent cover of encrusting bryozoans and *Ciona intestinalis* observed on kelp at six depths in Skagerrak in fall. Medians are represented by the horizontal line in each box, and the boxes comprise the first and third quartiles of each data group. Whiskers extend to the extreme data point which is no more than 1.5 times the interquartile range from the box. Recorded values that fall outside this range are represented by dots

In fall (Skagerrak only), the situation had changed dramatically, and all remaining SCK and WCK at all depths were covered with bryozoans (Figure 9). On kelp that had been kept at depths ranging from 1 to 9 m, one side (probably the side that had been facing upwards) was covered by vase tunicates and filamentous algae. The percentage cover of bryozoans and vase tunicates was relatively easy to assess, while the cover of epiphytic algae was a bit more difficult since the algae tended to grow in‐between the tunicates. The average densities of algal epiphytes found on kelps sampled in situ at 6 m depth in October were, however, 0.03 g/cm^2^. At 15 m depth, the densities of epibionts were significantly scarcer (Figure 9, Table 5). The statistical analysis also showed that there was a positive relationship between the presence of bryozoans and vase tunicates (see Table 5). The bryozoans form crusts on which vase tunicates may grow, while bryozoans never covered the vase tunicates.

**Table 5:** Analysis of epibiont coverage. Estimates from the analysis of coverage on *Saccharina latissima* survivors in Skagerrak in fall (percentages). The baseline (Intercept) in both binomial models was percent cover of epibionts on individuals from the west coast sample population. Station was included as a random factor influencing both the intercept and the slope of the models

Parameter	Estimate	*SE*	*z*	*p*
Vase tunicates				
Intercept	−0.937	2.404	−0.390	0.697
Pop (SCK vs. WCK)	−1.330	0.736	−1.808	0.070
Depth	−0.303	0.081	−3.739	≪0.001
Bryozoan coverage	4.638	2.380	1.949	0.051
Bryozoans				
Intercept	0.550	1.843	0.298	0.765
Pop (SCK vs. WCK)	−1.320	0.782	−1.688	0.092
Depth	−0.068	0.069	−0.983	0.326
Tunicate coverage	8.345	4.215	1.980	0.048

Kelps sampled in Skagerrak at approximately 6 m depth in October were also heavily fouled. The fronds were covered by a mosaic of epibiont very similar to those found on the rig individuals.

### 3.5 Shading by epibionts

**Figure 10:**
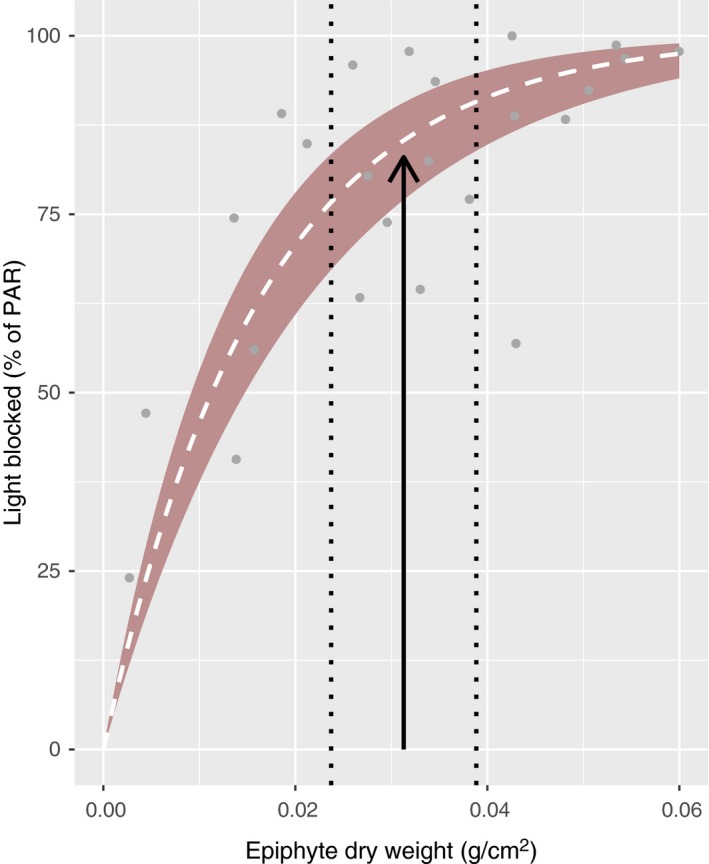
Shading by algal epiphytes. The vertical arrow represents the mean dry weight of algal epiphytes (g/cm^2^) found naturally occurring on *Saccharina latissima* in Skagerrak in October. The vertical dotted lines represent the 95% confidence interval of this mean. The relationship between epiphyte density (*X*) and light blocking (*Y*) is described by the model, *Y* ~ 100(1 − *e* ^−61.4^ *^X^*), and represented by the dashed line (white). The colored area depicts the 95% confidence interval of the model predictions. Data are shown as gray dots

The shading caused by epibiont coverage was substantial. Bryozoans appeared to be the least light depriving epibiont form, and the light reduction caused by a single layer of encrusting bryozoans was 11% (95% confidence interval [1%–59%], binomial distribution). Far more light, averagely 91%, was deprived by a single layer of vase tunicates (*C. intestinalis*; 95% confidence interval [35%–99%], binomial distribution). Increasing the densities of epiphytic algae (DW/cm^2^) caused rapid increases in shading (Figure 10), and the light reduction was well described by an exponential decay function (*t* = −8.884, *p* < 0.001, RSS = 0.479). The naturally occurring epiphyte density in October (not sampled from kelp on rigs, but from the kelp at the sampling site) reduced the light availability by averagely 85% according to this model (Figure 10).

## 4 DISCUSSION

The present study showed that kelp from the west coast (WCK) and the Skagerrak populations (SCK) responded similarly to the depth treatment and seasonal changes in the environment while mounted on rigs. Secondly, it indicates that that the light deprivation caused by epibionts may become lethal. Beyond that, further studies are needed in order to determine how epibionts impact natural populations, and how the impact varies with depth on a larger scale. That said, the following discussion supports the hypothesis that the recovery of *S. latissima* forests in Skagarrak is hindered by high levels of environmental stress and that fouling is likely to be an important stressor.

### 4.1 Growth and survival in the sample populations

The growth rates were quite high and did not differ among the two study sites in spring and summer, while poor growth and high mortality were recorded in Skagerrak in fall. Information about differences between the study sites in fall would have been valuable, but the rigs on the west coast were unfortunately lost.

Growth was consistently slower in SCK as compared to in WCK, but care must be taken in the interpretation of this result. Growth was measured as relative areal increase, and there are morphological differences between the west coast and the Skagerrak populations which leads to differences in biomass per thallus area. *S. latissima* individuals from Skagerrak have thicker laminas and appear sturdier than the individuals from the west coast (personal observations). Direct comparisons of growth rates as a measure of differences in production of new tissue may therefore be inaccurate.

We were, however, more interested in the effect depth had on the growth rates. The statistical analyses showed that the interaction between sample population and depth was not significant (Table 2), which indicate that the depth‐related growth response in the two populations was approximately the same. The consistency in responses (both growth and survival) between WCK and SCK in Skagerrak suggests that both sample populations were equally poorly adapted to handling the depth‐related stressors present at the Skagerrak site.

### 4.2 Depth‐related patterns of growth and survival

The depth limit of *S. latissima* is controlled by light availability, and a considerable upwards change in Skagerrak has been imputed to increased water turbidity (Rueness & Fredriksen, 1991; Walday et al., 2008). Recent surveys have documented that the depth distribution of *S. latissima* patches stops at 15 m depth in the northeastern part of Skagerrak (Moy & Christie, 2012, and references therein). Even though survival was good at 15 m depth in our study, low‐light conditions may have posed a challenge had we continued the experiment through the winter. Although *S. latissima* in arctic areas are able to endure very low‐light conditions (Borum, Pedersen, Krause‐Jensen, Christensen, & Nielsen, 2002), we do not know how the populations along the Norwegian mainland respond to similar conditions.

In order to estimate the success of a photosynthesizing species in a given habitat, growth is ecologically significant because it integrates many physiological processes, of which photosynthetic activity is very important (Bartsch et al., 2008). The organism has to maintain a positive carbon budget in order to grow, and when the consumption (i.e., respiration) increases, more light and/or more efficient photosynthesis is needed. The growth rates in individuals from both sample populations slowed down with increasing depth in spring and summer. This pattern was expected as a consequence of reduced light availability. Contrastingly, faster growth was observed at 15 m as compared to at shallower depths in fall. High temperatures reduce the kelps net photosynthesis (Sogn Andersen et al., 2013), and higher temperatures in shallow waters could have explained the slower growth. However, the light intensities recorded at 3 and 6 m depth should have been sufficient to support photosynthetic gain in *S. latissima* (Figure 7), and the sea water temperatures were not particularly high in 2009 (Figure 6). Low growth and high mortality at shallow depths may therefore also have been caused by other factors. The densities of epibionts in fall were very high close to the surface, and epibiosis is very likely to have multiple negative impacts on kelp growth and survival.

### 4.3 Effects of epibiont fouling

Epibionts have been shown detrimental to canopy forming marine macrophytes in other areas of the world (Saunders & Metaxas, 2008; Saunders, Metaxas, & Filgueira, 2010; Scheibling & Gagnon, 2009). Poor conditions of kelp in forest patches and high kelp mortality have coincided with fouling in Skagerrak (Moy & Christie, 2012; Sogn Andersen et al., 2011), suggesting that fouling organisms may have negative impacts in this area as well.

Epibionts deprive their host of light through shading, and reduced light availability may cause energy deficiency in photosynthesizing organisms like kelp. Our study showed that the extent of shading caused by ascidians and algae was considerable, while the shading caused by bryozoans was relatively modest. However, a positive relationship between bryozoan and vase tunicate coverage on the kelp frond suggested that bryozoan crusts may modify the kelp surface and facilitate settlement of other, more light absorbant species.

The kelp individuals monitored from 1 to 9 m depth in the rig study were densely covered by vase tunicates in fall, and the laboratory study showed that this may have deprived the individuals of as much as 90% of the available light. The difference in light intensity between 3 and 15 m depth in fall was also on average around 90%. Thus, the heavily fouled kelp plants located at 3 m depth may have received light amounts similar to the clean kelp plants at approximately 15 m depth. Growth was slower and mortality much higher at 3 m as compared to 15 m depth in fall, which may indicate either that the epibionts deprived the kelp of more light than estimated or that other factors than light influenced our results. Higher temperature at 3 m depth may have reduced the photosynthetic gain in the kelp, causing slower growth, and/or the epibionts may have had additional negative impacts on *S. latissima*.

In addition to blocking light, the presence of epibionts is likely to have negative impacts by increasing the diffusion boundary layers of their host (Jones, Eaton, & Hardwick, 2000; Sand‐Jensen, Revsbech, & Jörgensen, 1985) and form physical barriers that hamper nutrient uptake from the water (Hepburn et al., 2006). It has, however, been suggested that ammonium excretes from epibionts may serve as a nitrogen source for kelp during nutrient depletion, but these results are not conclusive (Hepburn & Hurd, 2005; Hepburn et al., 2006; Hurd, Durante, Chia, & Harrison, 1994; Hurd, Durante, & Harrison, 2000). In general, epibionts are likely to reduce the nutrient and carbon inflow from the surrounding sea water, and nutrient and carbon limitations will affect the growth of photosynthesizing hosts negatively. This could also explain the slow growth of heavily fouled kelp. Because nitrogen nutrition has been coupled to heat tolerance in *S. latissima* (Gerard, 1997), a reduction of the kelps resilience against heat stress may be a particularly relevant consequence. If epibionts reduce heat tolerance of their host, and their presence is most extensive in shallow waters, one might expect a downward push in the upper growth limit of *S. latissima* farther than predicted from temperature studies performed on clean kelp (i.e., most studies). The accumulation of epibionts may also harm host tissue (Hepburn et al., 2006) and increase the brittleness of the lamina, which results in increased erosion and defoliation during mechanical disturbance (Lee & Brinkhuis, 1988; Levin et al., 2002; Saunders & Metaxas, 2007; Scheibling & Gagnon, 2009).

The present study does not fully answer the question of how epibionts affect *S. latissima*, but it does suggest that the effect of epibionts should be incorporated into future research dealing with the distribution of kelp forests. Though it could be argued that the rig treatment may have affected the development of epibiont communities, procedural control in another study showed that dislodgement and translocation of kelp did not affect epibiont coverage (Marzinelli, Zagal, Chapman, & Underwood, 2009). As epibiont settlement in the present study occurred several months after the translocation (in concurrence with Sogn Andersen et al., 2011) and is commonly found on *S. latissima* in situ (Moy & Christie, 2012), we consider the coverage observed on translocated individuals to be the effect of location rather than the methods applied. Finally, the densities of algal epiphytes reported from the laboratory study were measured on kelp individuals sampled in the field in October, and these samples had not been subjected to rig treatment at all.

### 4.4 Final remarks

The WCK and the SCK sample populations did not exert different responses in relation to neither the depth treatment nor seasonal changes, they were equally fouled by epibionts and showed similar patterns of survival when exposed to the same environment. The regional difference in kelp forest recovery seems therefore most likely caused by environmental differences between the areas.

Both growth and survival of *S. latissima* in Skagerrak are likely to be reduced by heavy loads of epibionts. And while epibionts have severe impacts on kelp in shallow areas, especially during warm periods in summer and fall, depths where epibionts are sparse (i.e., around 15 m) may be close to the lower limit of the kelps depth distribution in this area. This suggests that a vertical squeeze, or narrowing of the distribution range of *S. latissima* may be occurring in Skagerrak. Epibionts are sparser and the distribution of *S. latissima* spans into deeper waters on the west coast (Trannum et al., 2012), which may explain why kelp forests in this area are seemingly more stable than in Skagerrak. Large‐scale and long‐term studies of natural populations are, however, needed, in order to test these hypotheses.

Although the kelp growth model was deemed appropriate for the purpose of the present discussion, it should not be applied for further predictions. Depth was used as a proxy for an intricate web of interactions of which epibiont densities, light, and temperature are important contributing factors, all of which vary on geographical scales.

## CONFLICT OF INTEREST

None declared.

## AUTHOR CONTRIBUTIONS

GSA, FEM, and HCH conceived the ideas, designed the study, and collected the data. GSA analyzed the data. GSA led the writing of the manuscript. All authors contributed to the drafts and gave final approval for publication.

## Data Availability

Datasets and R‐scripts are publicly available on GitHub (https://github.com/kelprmy/In-a-squeeze) and Dryad (Provisional https://doi.org/10.5061/dryad.k2r8410).
